# Protopine Alleviates Dextran Sodium Sulfate-Induced Ulcerative Colitis by Improving Intestinal Barrier Function and Regulating Intestinal Microbiota

**DOI:** 10.3390/molecules28135277

**Published:** 2023-07-07

**Authors:** Meishan Yue, Jialu Huang, Xiaolan Ma, Peng Huang, Yisong Liu, Jianguo Zeng

**Affiliations:** 1 College of Veterinary Medicine, Shanxi Agricultural University, Jinzhong 030801, China; 18404984883@163.com (M.Y.); hhjj8181@163.com (J.H.); mxlmxl1997@163.com (X.M.); 2 College of Veterinary Medicine, Hunan Agricultural University, Changsha 410128, China; huangpeng@hunau.edu.cn (P.H.); liuyisong@hunau.edu.cn (Y.L.)

**Keywords:** protopine, ulcerative colitis, intestinal microbiota, intestinal mucosal barrier

## Abstract

Ulcerative colitis (UC) is an inflammatory bowel disease (IBD), and its pathogenesis is related to intestinal mucosal barrier damage and gut microbiota imbalance. Protopine (PRO), an isoquinoline alkaloid, is one of the main anti-inflammatory ingredients of traditional Chinese medicine *Macleaya cordata* **(Willd.) R. Br**. This study investigated the effects of PRO on the intestinal mucosal barrier and gut microbiota in dextran sodium sulfate (DSS)-induced colitis mice. C57BL/6J mice were treated with 3% DSS in drinking water to induce acute colitis, while PRO was administered orally once daily for 7 days. The results showed that PRO administration significantly alleviated the symptoms of DSS-induced colitis in mice and inhibited the expression of inflammation-related genes. In addition, PRO restored the integrity of the intestinal barrier in colitis mice by restoring colonic mucin secretion and promoting the expression of tight junction proteins. Furthermore, PRO alleviated the DSS-induced gut microbiota dysbiosis by decreasing the abundance of *Proteobacteria*, *Escherichia-Shigella* and *Enterococcus*, as well as enhancing the abundance of beneficial bacteria, such as *Firmicutes* and *Akkermansia*. These findings suggested that PRO effectively alleviated DSS-induced ulcerative colitis by suppressing the expression of inflammation-related genes, maintaining the intestinal mucosal barrier and regulating the intestinal microbiota.

## 1. Introduction

The intestine is a key organ for critical physiological functions such as nutrient absorption, metabolism, defense and immunity. The intestines of animals are highly susceptible to factors such as environment and diet, which lead to intestinal inflammation and a series of health problems such as diarrhea. Diarrhea can reduce animal production performance and even lead to animal death, causing economic losses to the livestock and poultry breeding industries. Animal inflammatory bowel disease (IBD) is a chronic non-specific inflammatory disease of the gastrointestinal tract that endangers animal and human health. Ulcerative colitis (UC), a kind of IBD, is defined by persistent inflammation of the colonic mucosa and submucosa, with clinical manifestations of diarrhea, abdominal pain and bloody stools [1].

Despite the complexity of the pathogenesis of UC, studies have demonstrated that immune system disorders, intestinal mucosal barrier damage and disturbances in the intestinal microbiota contribute to UC occurrence [2,3,4]. The disruption of intestinal mucosal barrier integrity increases bacterial antigen translocation, and innate immune cells are stimulated by antigens to activate the nuclear factor kappa B (NF-κB) pathway through Toll-like receptors, thereby activating the transcription of pro-inflammatory factors and mediators, such as interleukin-6 (IL-6), interleukin-1 beta (IL-1β), and tumor necrosis factor-alpha (TNF-α), inducible nitric oxide synthase (iNOS) and cyclooxygenase-2 (COX-2) [5]. The excessive secretion of inflammatory cytokines and mediators aggravates the inflammatory cascade reaction, further causing colon injury and forming a vicious circle [6]. The mucus layer, intestinal epithelial cells (IECs) and tight junctions (TJs) work together against pathogen invasion and assist the organism in supporting the intestine’s integrity [7]. The gut microbiota is involved in various functions, such as host immune regulation and energy metabolism, and is essential for preserving host health. Moreover, increasing evidence indicates that gut microbiota is also commonly associated with intestinal inflammation [8,9]. Dysregulation of gut microbiota can lead to decreased bacterial diversity, increased intestinal permeability and pathogenic bacteria, aggravating the inflammatory process [10,11]. Protecting the stability of the intestinal mucosal barrier and intestinal flora is of great significance to intestinal health.

The main drugs currently used to treat UC in humans contain aminosalicylic acids, glucocorticoids, immunosuppressants and other chemically synthesized drugs. Animal UC is mainly treated with antibiotics but is prone to antibiotic abuse, leading to microbial resistance. Therefore, it is essential to screen effective drug candidates from natural plant-derived bioactive substances.

*Macleaya cordata* **(Willd.) R. Br.**, a member of the *Papaveraceae* family, is a traditional Chinese herb with anti-inflammatory, anti-bacterial, anti-oxidant, insecticidal and anti-tumor effects [12,13,14]. Its main functional active ingredients are isoquinoline alkaloids such as sanguinarine, chelerythrine, protopine, and allocryptopine. Bopu powder, a product mainly composed of protopine and allocryptopine, has been developed in China as an animal medication (Veterinary Drug No. 180415374) for treating chicken diarrhea caused by *Escherichia coli*. It is reported that adding *Macleaya cordata* extract, which consists of sanguinarine and chelerythrine, to the food can improve animal immunity, production performance and intestinal health [15,16]. Protopine (PRO) has been shown to exhibit many biological effects, including anti-inflammatory, anti-thrombotic, anti-oxidant, anti-tumor, analgesic and liver protection [17,18,19]. Previous studies have shown that PRO inhibits apoptosis and inflammation through the TLR4 signaling pathway, thereby protecting mice from LPS-induced AKI [20]. Furthermore, PRO reduces the inflammatory responses caused by CA and LPS via modulating MAPK/NF-κB signaling [21,22]. In addition, PRO stimulates HCT116 cell apoptosis and autophagy by activating the p53 pathway [23]. Recent studies have found that PRO reduces inflammation and oxidation stress by suppressing NLRP3 and NF-κB signaling pathways, thereby decreasing NCM460 colon epithelial cell damage caused by LPS [24].

Although PRO has many of the above-mentioned biological activities, its function in dextran sodium sulfate (DSS)-induced ulcerative colitis is unknown. Therefore, this study established a mouse UC model induced by DSS and explored the influence of PRO on the colonic mucosal barrier and intestinal flora in mice with colitis.

## 2. Results

### 2.1. PRO Attenuated the Symptoms of DSS-Induced Colitis in Mice

**Figure 1:**
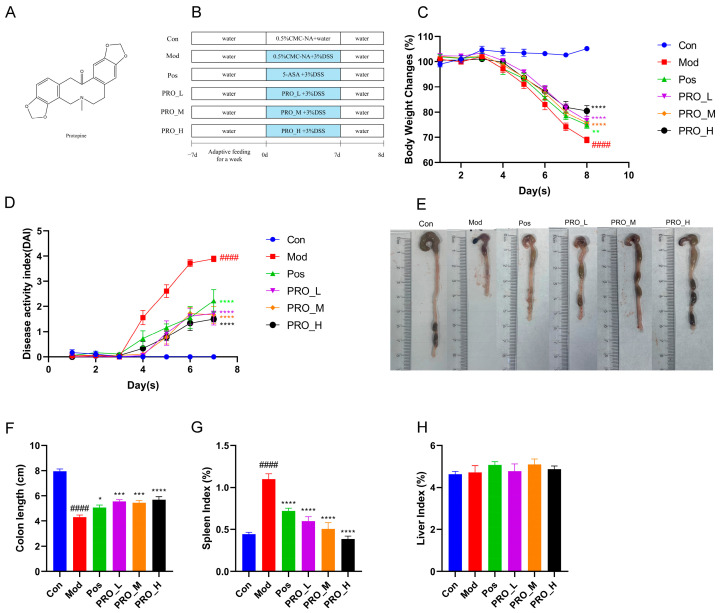
The effect of PRO on DSS-induced colitis in mice. (**A**) The structure of Protopine (PRO). (**B**) Schematic of animal experimental procedures. (**C**) Changes in body weight of mice in each group. (**D**) The disease activity index (DAI) scores of mice in every group. (**E**,**F**) Representative images and colon length of each group. (**G**) Spleen index and (**H**) Liver index of each group. Data are presented as the mean ± SEM (*n* = 6). #### *p* < 0.0001 vs. Con group; * *p* < 0.05, ** *p* < 0.01, *** *p* < 0.001, and **** *p* < 0.0001 vs. Mod group. Note: Con = control group; Mod = model group; Pos = positive group; PRO_L = The low dose of PRO group; PRO_M = The medium dose of PRO group; PRO_H = The high dose of PRO group.

Compared with the Con group, the Mod group mice showed weight loss, significantly increased DAI scores, and greatly shortened colon length (Figure 1C–F), suggesting that a mouse model of UC was successfully constructed. By contrast, compared to the Mod group, supplementation with PRO and 5-ASA significantly inhibited DSS-induced weight loss and increased DAI scores in mice and resulted in a significant increase in colon length. In addition, DSS administration significantly increased the Mod group mice’s spleen index, which was significantly attenuated by PRO and 5-ASA treatment (Figure 1G). The liver index of mice did not significantly change among the groups (Figure 1H). 

**Figure 2:**
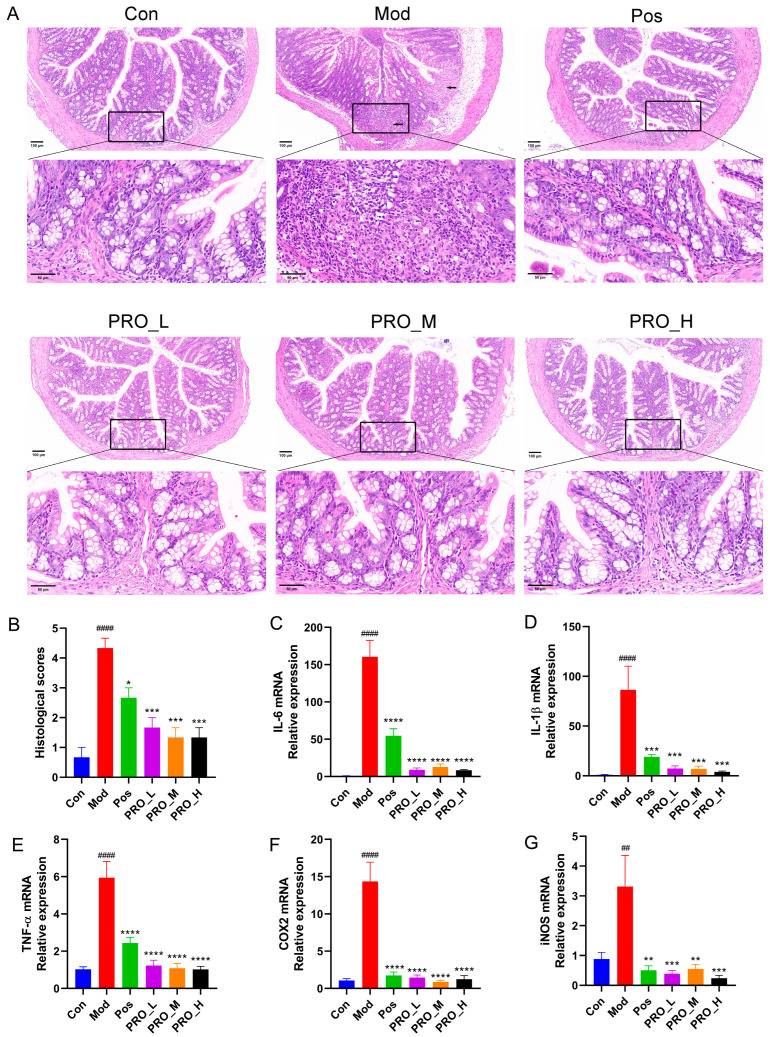
The effect of PRO on histological damage and colon inflammation in mice with DSS-induced colitis. (**A**) Representative images of hematoxylin and eosin (HE) staining of colon sections. (Magnification at ×400, scale bar 50 μm, arrows-ulceration). (**B**) Comparison of histological scores between groups. (**C**–**G**) The mRNA expression of inflammation-related genes IL-6, IL-1β, TNF-α, COX2 and iNOS in colon tissue. Data are presented as the mean ± SEM (*n* = 4). ## *p* < 0.01, #### *p* < 0.0001 vs. Con group; * *p* < 0.05, ** *p* < 0.01, *** *p* < 0.001 and **** *p* < 0.0001 vs. Mod group. Note: Con = control group; Mod = model group; Pos = positive group; PRO_L = The low dose of PRO group; PRO_M = The medium dose of PRO group; PRO_H = The high dose of PRO group.

In this study, HE staining was performed to further evaluate the colonic pathological damage. As shown in Figure 2A, the intestinal epithelium of mice in the Con group was complete and continuous, with neatly arranged goblet cells and no infiltration of inflammatory cells. In the Mod group, the intestinal epithelial cells were seriously disordered, some crypts and goblet cells disappeared, local mucosal ulcers were observed, and the submucosa was edematous and infiltrated by many inflammatory cells. After treatment with PRO and 5-ASA, the inflammation of the colonic mucosa was relieved, the epithelium was more intact, and the histological scores were significantly reduced (Figure 2B).

### 2.2. PRO Inhibited the Expression of Inflammation-Related Genes in DSS-Induced Colitis

The abnormal increase in inflammatory cytokines and mediators leads to a prolonged inflammatory response, which is related to the pathogenesis of UC. In this study, to assess the anti-inflammatory effect of PRO, qRT-PCR was used to detect the mRNA levels of inflammatory cytokines and mediators in colon tissue. TNF-α, IL-1β, IL-6, COX2 and iNOS mRNA levels were significantly upregulated in the Mod group compared to the Con group. At the same time, treatment with PRO and 5-ASA effectively reversed these upregulations (Figure 2C–G).

### 2.3. PRO Ameliorated Colonic Barrier Dysfunction in Mice with DSS-Induced Colitis

**Figure 3:**
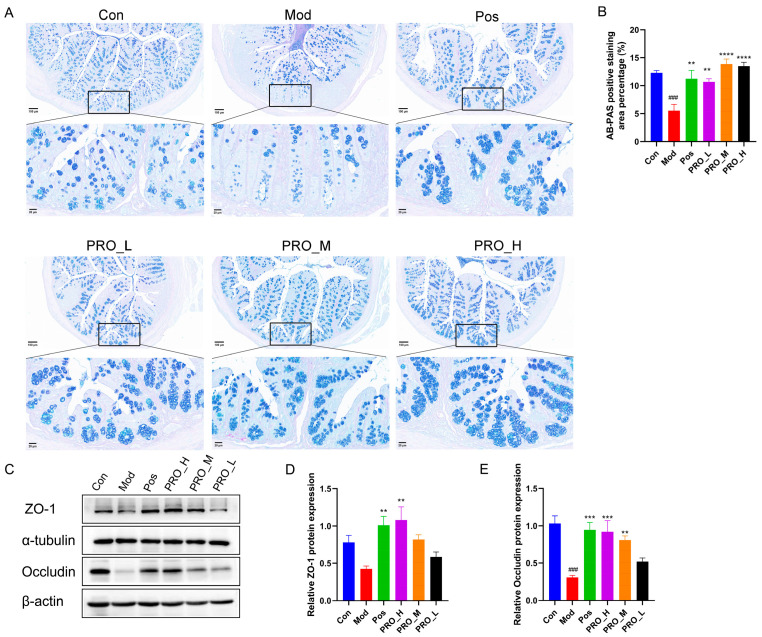
The effect of PRO on mucosal barrier integrity in colitis mice. (**A**) Representative images of Alcian blue-periodic acid Schiff (AB-PAS) staining of colon sections. (Magnification at ×400, scale bar 20 μm). (**B**) The percentage of AB-PAS positive staining area. (**C**) Representative immunoblots for the tight junction proteins zonula occludens 1 (ZO-1) and Occludin. The relative expression of ZO-1 (**D**) and Occludin (**E**). Data are presented as the mean ± SEM (*n* = 4). ### *p* < 0.001 vs. Con group; ** *p* < 0.01, *** *p* < 0.001, and **** *p* < 0.0001 vs. Mod group. Note: Con = control group; Mod = model group; Pos = positive group; PRO_L = The low dose of PRO group; PRO_M = The medium dose of PRO group; PRO_H = The high dose of PRO group.

AB-PAS staining can stain the acidic mucin blue and the mixed mucin blue-purple. The results showed that in Figure 3A, goblet cells in the Con group were numerous and secreted many mucins. Goblet cells in the Mod group were damaged and mucin secretion was significantly reduced compared to the Con group. However, PRO and 5-ASA treatment alleviated the reduction in goblet cells and significantly increased mucin content (Figure 3B). In addition, expression of ZO-1 and Occludin in colonic tissues was detected by Western Blot. Although the expression of ZO-1 and Occludin in the Mod group was decreased relative to the Con group, both PRO and 5-ASA treatment elevated ZO-1 and Occludin protein levels to different degrees (Figure 3C–E).

### 2.4. PRO Modulated Gut Microbiota Dysbiosis in DSS-Induced Colitis Mice

**Figure 4:**
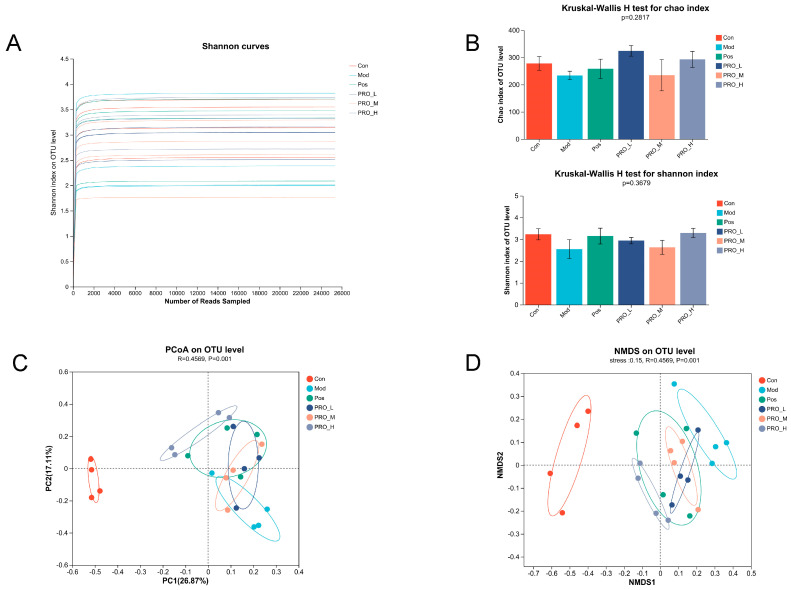
The effect of PRO on α diversity and β diversity of gut microbiota in colitis mice caused by DSS. (**A**) The Shannon curve. (**B**) The α diversity (Chao index and Shannon index). (**C**) The PCoA analysis for β diversity. (**D**) The NMDS analysis for β diversity. *n* = 4 for each group. Note: Con = control group; Mod = model group; Pos = positive group; PRO_L = The low dose of PRO group; PRO_M = The medium dose of PRO group; PRO_H = The high dose of PRO group.

In Figure 4A, the Shannon curve trended smoothly, indicating that there was enough sequencing data to capture most of the microbial diversity information of the samples. Microbial α-diversity in this study was reflected by the Chao and Shannon index, which did not vary significantly among the groups (Figure 4B). PCoA analysis was conducted to study the similarity or differences in microbial community composition. The gut microbiota of the Mod group was separated from that of the Con group (Figure 4C), suggesting that DSS treatment induced an intestinal flora imbalance. The microbiota of the PRO and 5-ASA treatment groups were closer to the Con group than the Mod group, indicating that PRO showed a protective function against DSS-induced dysbacteriosis. Furthermore, we obtained similar results in NMDS (Figure 4D).

**Figure 5:**
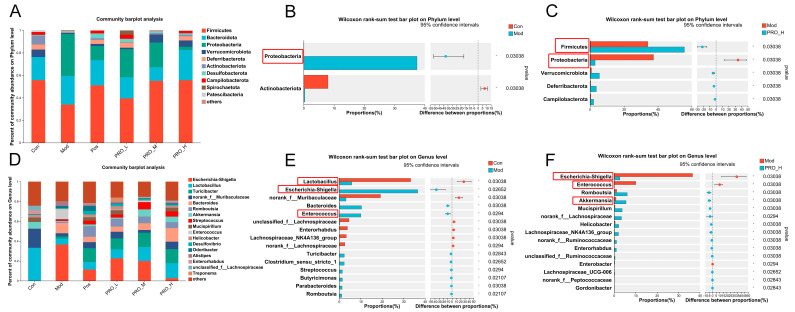
The effect of PRO on the composition of intestinal microflora in colitis mice induced by DSS. (**A**) Percent of community abundance on phylum level. (**B**) Wilcoxon rank-sum test bar plot on phylum level between Con and Mod groups. (**C**) Wilcoxon rank-sum test bar plot on phylum level between Mod and PRO_H groups. (**D**) Percent of community abundance on genus level. (**E**) Wilcoxon rank-sum test bar plot on genus level between Con and Mod groups. (**F**) Wilcoxon rank-sum test bar plot on genus level between Mod and PRO_H groups. * *p* < 0.05. Note: Con = control group; Mod = model group; Pos = positive group; PRO_L = The low dose of PRO group; PRO_M = The medium dose of PRO group; PRO_H = The high dose of PRO group.

We then examined the bacterial taxonomic analysis at the phylum and genus levels. And based on the community abundance data in the sample, the significant difference test between groups was carried out to evaluate the significant level of species abundance difference. The Wilcoxon rank sum test does not require sample size or variance and is mainly used as a method for the non-parametric testing of two independent samples. This study used the Wilcoxon rank sum test for pairwise comparison. At the phylum level, DSS stimulation in the Mod group greatly lowered the level of *Actinobacteriota* and increased the level of *Proteobacteria* compared to the Con group. In contrast, high-dose PRO treatment corrected the content of *Proteobacteria* changed by DSS. In addition, the levels of *Firmicutes*, *Verrucomicrobiota*, *Deferribacterota* and *Campilobacterota* in the PRO-H group were substantially increased compared to the Mod group (Figure 5A–C). At the genus level, the Mod group showed a marked decrease in beneficial bacteria, such as *Lactobacillus,* compared to the Con group. While *Escherichia-Shigella*, *Enterococcus*, *Streptococcus* and other pathogenic bacteria were heavily enriched. In comparison to the Mod group, the high-dose PRO intervention greatly lowered the percentage of *Escherichia-Shigella* and *Enterococcus*, in addition, the content of *Akkermansia* and other beneficial bacteria was greatly increased (Figure 5D–F).

**Figure 6:**
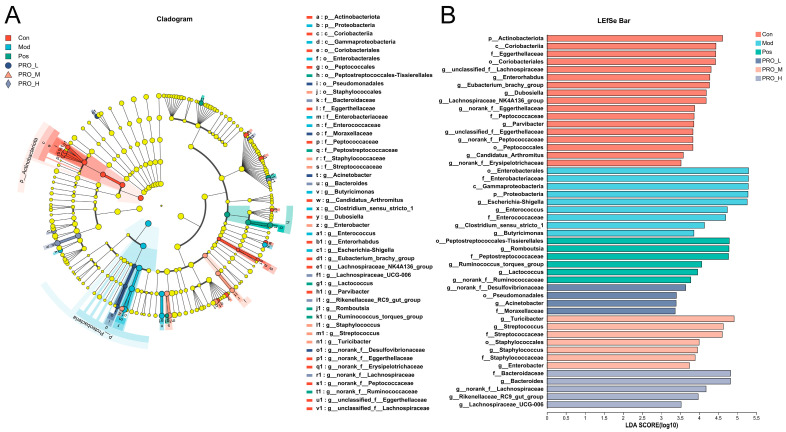
The LEfSe analysis of microbial abundance among groups. (**A**) Taxonomic cladogram of bacteria from all groups. (**B**) LDA scores of bacterial taxa that were significantly enriched in each group (LDA score > 2). Note: Con = control group; Mod = model group; Pos = positive group; PRO_L = The low dose of PRO group; PRO_M = The medium dose of PRO group; PRO_H = The high dose of PRO group.

The representative species of each group were further determined by LEfSe analysis (Figure 6). Based on an LDA threshold greater than two, we found that Phylum *Actinobacteriota* was enriched in the Con group. The Mod group had a high abundance of opportunistic pathogens such as Phylum *Proteobacteria*, genera *Escherichia-Shigella* and genera *Enterococcus*. And some potential beneficial bacteria, such as genera *Romboutsia*, genera *Lactococcus* and genera *Bacteroides,* were enriched in the Pos and PRO_H groups.

### 2.5. Correlation Analysis between Inflammation-Related Genes and Intestinal Microbiota 

**Figure 7:**
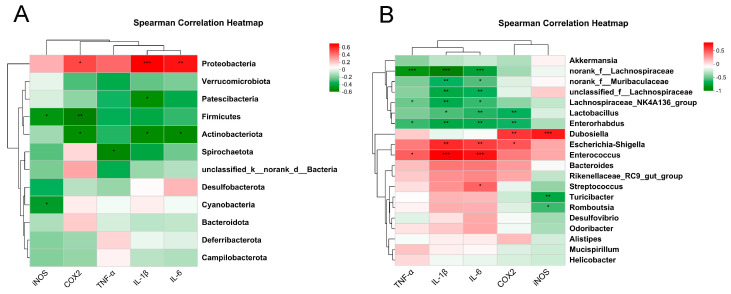
Spearman correlation analysis between intestinal microflora and inflammatory cytokines. (**A**) Phylum level. (**B**) Genus level. * *p* < 0.05, ** *p* < 0.01 and *** *p* < 0.001.

Spearman’s rank coefficient of correlation is a non-parametric test method used to measure the strength of the relationship between variables. It does not require the assumption of normality and equal variance of variables and has a wide range of applications. To investigate the potential relationship between altered gut microbiota and inflammation-related genes, we performed a correlation analysis using Spearman’s rank coefficient of correlation at both the phylum and genus levels. The levels of IL-1β, IL-6 and COX2 were positively correlated with *Proteobacteria* and negatively related to *Actinobacteriota* at the phylum level. *Firmicutes* were negatively associated with the levels of iNOS and COX2 (Figure 7A). By analyzing the genus level, pathogenic bacteria, including *Escherchia-Shigella*, *Enterococcus* and *Streptococcus*, were positively related to the levels of inflammation-related genes. In contrast, *Lactobacillus* and other beneficial bacteria were negatively related to the expression of inflammation-related genes (Figure 7B).

## 3. Discussion

Increasing plant-derived bioactive alkaloids have been shown to participate in regulating the development of IBD [25,26]. For instance, sanguinarine relieved DSS-induced experimental colitis by blocking the NLRP3 pathway and improving intestinal microbial disorders [27]. Additionally, allocryptopine improved the intestinal barrier and reduced the colitis response by inhibiting CX3CL1/GNB5/AKT2/NF-κB/apoptosis pathway [28]. PRO is an important isoquinoline alkaloid with strong pharmacological activity and plays a key regulatory function in a variety of diseases [19,20], but its role in intestinal inflammation remains unknown. This research showed that PRO could significantly reduce the severity of ulcerative colitis, decrease injuries to the colonic barrier, and improve intestinal microbial disorders, revealing the therapeutic potential of PRO in UC.

This study successfully established a mouse model of acute colitis by giving mice 3% DSS through drinking water for 7 days. In agreement with earlier studies [25,27], we discovered that DSS-induced colitis mice showed the characteristics of decreased body weight, bloody stools and shortened colon. In addition, the spleen index increased, which is usually related to the severity of inflammation. However, PRO treatment could significantly alleviate these colitis symptoms. The damage to the colonic mucosa was determined using HE staining in this research. PRO could significantly alleviate the damage to the colon tissue, which was manifested by reducing inflammatory cell infiltration and crypt destruction and making the mucosal epithelium more complete. Furthermore, the release of different pro-inflammatory cytokines, like IL-6, IL-1β and TNF-α, is a significant signal for the enhancement and continuation of UC [29]. As well as the activation of inflammatory mediators like iNOS and COX-2 has been shown to induce intestinal injury in colitis [29]. In this experiment, PRO effectively down-regulated the expression levels of these inflammatory factors and mediators, which agreed with the findings of earlier studies [21,22]. We speculated that PRO might inhibit DNA strand binding or cause altered or lost RNA polymerase activity, thereby interfering with the transcription process, inhibiting mRNA synthesis of inflammatory factors, and attenuating the inflammatory response. Therefore, these results preliminarily confirmed that PRO could inhibit the levels of inflammatory markers and reduce the symptoms of colitis in mice.

Intestinal mucosal barrier dysfunction has been reported to be frequently associated with UC [2,25,30]. Colonic mucus and intercellular TJs collaborate to form a highly integrated barrier system that is essential for intestinal defense [31]. The goblet cells and their secreted mucins constitute the intestinal mucus layer, which serves as the body’s first line of protection against microorganisms and pathogens. Reduced or abnormal mucus secretion leads to bacterial and pathogen invasion and excessive inflammation, contributing to the development of UC [32]. AB-PAS staining was performed in this work to assess the growth of goblet cells and mucin secretion. As expected, our data suggested that PRO treatment increased the number of goblet cells in the colon of mice with colitis and improved mucus secretion significantly. Moreover, the scaffold protein zonula occludens 1 (ZO-1) and the transmembrane protein Occludin are the main proteins that constitute TJs. Studies have shown that decreased expression of TJ proteins in IBD can result in a rise in intestinal permeability [33]. The present findings were consistent with previous reports that DSS induction decreased TJ protein levels [25,30]. Also, we found that PRO increased ZO-1 and Occludin protein levels in the colon after DSS injury, with a better effect at higher doses. Taken together, PRO could enhance mucin secretion and improve the levels of TJ proteins, hence protecting intestinal barrier integrity and alleviating UC in mice.

It is agreed that gut microbiota homeostasis is essential to host health [8,9]. The structure of the intestinal flora changed in UC patients and animal models, showing an imbalance between beneficial and harmful bacteria [30,34]. Some scholars have proposed that the continuous rise in the abundance of *Proteobacteria* is a potential diagnostic marker for inflammation and disease [35]. Consistent with these findings, our data also showed that the Mod group had a significantly higher percentage of *Proteobacteria*, and high-dose PRO could significantly reverse this phenomenon. At the same time, we observed that high-dose PRO significantly increased the content of *Firmicutes*. It has been shown that *Firmicutes* inhibits intestinal inflammation by regulating colonic pH and inhibiting pathogen growth [36]. In the present study, *Lactobacillus* relative abundance was considerably lower in the Mod group than in the Con group at the genus level, while pathogenic bacteria commonly associated with diseases like *Escherichia-Shigella* and *Enterococcus* were remarkably expanded. These results were similar to previous studies [37,38] and again demonstrated that DSS caused dysbiosis of the intestinal flora. *Lactobacillus* is a probiotic that inhibits the production of inflammatory cytokines, limiting harmful bacteria activity in the intestine and having a protective effect against IBD [39]. The *Lactobacillus* content recovered a little after high-dose PRO treatment, although it did not reach statistical significance. However, the levels of *Escherichia-Shigella* and *Enterococcus* were significantly reduced. Interestingly, we also found a significant enrichment of *Akkermansia* after high-dose PRO intervention. Several studies have identified *Akkermansia* as a bacterium colonized in the mucus layer and involved in the thickening of the mucus layer and enhancement of the gut barrier [40,41]. In this study, AB-PAS results revealed that mucin secretion increased significantly after PRO intervention. Given the positive effect of *Akkermansia* on intestinal mucosal integrity, we speculated that the repairing effect of PRO on intestinal mucosa might also be related to the enrichment of *Akkermansia*. Therefore, our study showed that PRO could effectively restore the composition of intestinal flora destroyed by DSS induction by inhibiting harmful bacteria growth and promoting the reproduction of beneficial bacteria.

During this experiment, Spearman’s rank correlation method revealed that the release of inflammatory cytokines and mediators was positively correlated with an increase in harmful bacteria and negatively associated with a reduction in beneficial bacteria. It is suggested that intestinal mucosal inflammation is closely related to alterations in intestinal flora. However, the causal relationship behind this correlation must be further studied.

In summary, PRO can effectively alleviate DSS-induced ulcerative colitis by inhibiting the expression of inflammatory factors, restoring intestinal barrier function and improving intestinal microbial imbalance. Of course, the exact mechanism by which PRO mitigates UC remains to be elucidated.

## 4. Materials and Methods

### 4.1. Animals and Experimental Design

Male C57BL/6J mice (aged 8 weeks, weighing 18–22 g) were obtained from Tianqin Biotechnology Co., Ltd. (Changsha, China) [license no. SCXK (Xiang) 2019-0014]. Mice had free access to water and food under experimental conditions that maintain a fixed 12 h light/dark cycle at 23 °C.

Mice were randomly assigned to the following six groups (*n* = 6) after one week of acclimatization: (1) Control group (Con): mice had access to pure water; (2) Model group (Mod): mice were given free access to 3% dextran sulfate sodium (DSS, MW: 36–50 KDa, MP Biomedicals, Santa Ana, CA, USA) for 7 days to induce colitis; (3) Positive group (Pos): mice were treated with 3% DSS solution and intragastrically administrated 200 mg/kg 5-aminosalicylic acid (5-ASA, purity > 98%, Lot: 01026A, Meilun Biotechnology, Dalian, Liaoning, China) once daily for 7 days; (4), (5) and (6) The low, medium and high dose of PRO groups (PRO_L, PRO_M, PRO_H): mice were treated with 3% DSS solution and intragastrically administrated PRO (25, 50 or 100 mg/kg, purity > 98%, Lot: 100801, the structure is shown in Figure 1A, Meikeda Biological Resources, Changsha, Hunan, China) once daily for 7 days. At the same time, Con and Mod mice were gavaged with an equal amount of 0.5% Carboxymethylcellulose sodium (CMC-NA). The experimental layout is shown in Figure 1B.

As previously mentioned [42], the weight loss, fecal consistency and rectal bleeding of mice were observed daily during the experiment and scored according to Table 1. The disease activity index (DAI) score = (weight loss score + fecal consistency score + rectal bleeding score)/3. After the mice were sacrificed, their colon length, spleen weight and liver weight were measured. Part of the colon tissue was fixed with 4% paraformaldehyde, and the rest was frozen in liquid nitrogen. Colon contents were kept at −80 °C for gut microbiota analysis.

### 4.2. HE and AB-PAS Staining

For histological observation, colon tissue was embedded in paraffin, sectioned and stained with hematoxylin and eosin (HE). Histological scoring was assessed as described by Erben et al. [43]. Alcian blue-periodic acid Schiff (AB-PAS) was used to assess the secretion of mucin in colon tissue. The percentage of AB-PAS positive staining area in the total area of the colonic epithelium was determined by Image Pro Plus 6.0.

### 4.3. Quantitative Real-Time Polymerase Chain Reaction (qRT-PCR) 

Total RNA from colon tissue was extracted and cDNA was synthesized and stored at −20 °C. qRT-PCR was conducted on a qTOWER^3^ (Analytik Jena, Jena, Germany) using the SYBR^®^ Green Premix Pro Taq HS qPCR Kit (Accurate Biology, Changsha, China). The relative mRNA expression of the target genes in every sample was determined using the 2^−ΔΔCt^ method. According to the mRNA sequence in the NCBI database, primers were designed by Primer 5.0 program and synthesized by Accurate Biology (Changsha, China). Table 2 displays each primer sequence individually.

### 4.4. Western Blot Analysis

Total protein was extracted, separated by SDS-PAGE gel, and transferred to the PVDF membrane. Membranes were blocked with 5% skim milk and incubated at 4 °C overnight with anti-Occludin (1:3500, 27260-1-AP, Proteintech, Wuhan, Hubei, China), anti-ZO-1 (1:1000, ab49602, Abcam, Cambridge, UK), anti-α-tubulin (1:50,000, 66031-1-Ig, Proteintech, Wuhan, Hubei, China) and anti-β-actin (1:50,000, AC026, Abclonal, Wuhan, Hubei, China). Membranes were washed with TBST, incubated with secondary antibodies, and then observed using an enhanced chemiluminescence kit. ImageJ was used to analyze the protein expression levels.

### 4.5. Gut Microbiota Analysis

Total DNA from mouse colon contents was extracted and the integrity of the DNA was detected with 1% agarose gel. The hypervariable region V3–V4 of the bacterial 16S rRNA gene was amplified by PCR. PCR products were cut and recovered using the AxyPrepDNA Gel Recovery Kit (AXYGEN Company, Union City, CA, USA). The PCR products were quantified by QuantiFluor™ ST Blue Fluorescence Quantitative System (Promega, Madison, WI, USA). The Illumina libraries were constructed and the 16S rRNA sequencing was performed on the Illumina Miseq platform, completed by Shanghai Majorbio Bio-Pharm Technology Co., Ltd. (Shanghai, China). After the sequencing data were spliced and quality-controlled, the optimized sequence was obtained. Based on the optimized sequence, operational taxonomic unit (OTU) clustering was performed by UPARSE (version 11) at 97% similarity, and taxonomic information of OTU representative sequences was obtained for subsequent bioinformatics analysis. Alpha diversity analysis was performed using Mothur software (version 1.30.2). Qiime (version 1.9.1) calculated the beta diversity distance matrix and then plotted it with the R software (version 3.3.1). The linear discriminant analysis (LDA) effect size (LEfSe) method was used to find species with significant differences in abundance between groups. All analyses were performed on the Majorbio cloud platform (www.majorbio.com accessed on 18 July 2022).

### 4.6. Statistical Analysis

Data analysis was performed using one-way ANOVA followed by Dunnett’s multiple comparison test using GraphPad Prism 8.0 (GraphPad Software, San Diego, CA, USA). The data were expressed as mean ± standard error of the mean (SEM). *p* < 0.05 was considered statistically significant.

## Figures and Tables

**Table 1:** The disease activity index (DAI) evaluation.

Scores	Weight Loss	Fecal Consistency	Rectal Bleeding
0	none	normal	no bleeding
1	1–5%		
2	5–10%	loose stools	slight bleeding
3	10–15%		
4	>15%	watery diarrhea	gross bleeding

**Table 2:** The primer sequences for qRT-PCR.

Gene Name	Primer Sequence (5′–3′)
IL-6	F:AGACTTCCATCCAGTTGCCT R:CAGGTCTGTTGGGAGTGGTA
IL-1β	F:ACTCATTGTGGCTGTGGAGA R:TTGTTCATCTCGGAGCCTGT
TNF-α	F:ATGTCTCAGCCTCTTCTCATTC R:GCTTGTCACTCGAATTTTGAGA
COX2	F:ATTCCAAACCAGCAGACTCATA R:CTTGAGTTTGAAGTGGTAACCG
iNOS	F:ATCTTGGAGCGAGTTGTGGATTGTC R:TAGGTGAGGGCTTGGCTGAGTG
β-Actin	F:CTGTGCCCATCTACGAGGGCTAT R:TTTGATGTCACGCACGATTTCC

Abbreviations: F, forward; R, reverse.

## Data Availability

The data presented in the study are deposited in the NCBI SRA BioProject repository, accession number (PRJNA977474).

## Sample Availability

Samples of the compounds are available from the authors upon request.
